# Evidence-based guidelines on orthobiologics

**DOI:** 10.1530/EOR-2025-0069

**Published:** 2025-06-02

**Authors:** Tobias Winkler, Stephan Oehme, Alexander Hildebrandt, Azzurra Paolucci, Lorenz Pichler

**Affiliations:** ^1^ Charité – Universitätsmedizin Berlin, Corporate Member of Freie Universität Berlin and Humboldt-Universität zu Berlin, Center for Musculoskeletal Surgery (CMSC), Berlin, Germany; ^2^ Berlin Institute of Health (BIH) at Charité – Universitätsmedizin Berlin, Julius Wolff Institute (JWI), Berlin, Germany; ^3^ Berlin Institute of Health (BIH) at Charité – Universitätsmedizin Berlin, BIH Center for Regenerative Therapies (BCRT), Berlin, Germany; ^4^Berlin Institute of Health (BIH) at Charité – Universitätsmedizin Berlin, BIH Biomedical Innovation Academy, BIH Charité Junior Clinician Scientist Program, Berlin, Germany; ^5^ Università degli Studi Alma Mater Studiorum di Bologna, IRCCS Istituto Ortopedico Rizzoli di Bologna, Bologna, Italy; ^6^ Medical University of Vienna, Department of Orthopedics and Trauma-Surgery, Vienna, Austria

**Keywords:** Orthobiologics, Regenerative Therapies, Advanced Therapies

## Abstract

- Orthobiologics (OBs) have seen a constant increase in the number of available therapies and their clinical applications. Existing therapies can be categorized into blood-based (e.g., platelet-rich plasma (PRP)) and tissue/cell-based (e.g. mesenchymal stromal cells) approaches. While the popularity of OBs continues to grow, their diverse natures create unique challenges for the establishment of evidence-based guidelines.
- PRP has been reported by meta-analyses to increase patient-reported outcomes for conditions such as knee osteoarthritis (KOA), lateral epicondylitis and plantar fasciitis. However, the randomized controlled trials (RCTs) included often exhibit a high risk of bias due to the heterogeneity in the PRP preparation protocols and accompanying measures as well as inconsistent trial quality.
- The development pipeline of cell/tissue-based therapies is typically longer and more cost-intensive than that of blood-based therapies. Nevertheless, several products have demonstrated clinical safety. While some RCTs and meta-analyses on the outcome of cell/tissue-based therapies exist, their number is considerably lower than that of blood-based therapies and they focus mainly on KOA, with limited evidence on other orthopedic indications.
- Orthopedic societies such as ESSKA and AAOS have taken on the challenge of developing guidelines for OBs by combining high-level synthesized evidence with expert consensus.
- Patient stratification strategies represent a promising key to unlocking the full potential of OBs and are currently being investigated in ongoing studies.
- Further efforts to establish guidelines for the use of OBs should focus on developing frameworks for clinical trials and their reporting, alongside standardized protocols for the preparation, application and accompanying measures of OB therapies.

## Introduction

The term ‘Orthobiologics’ (OB) is used for a wide and heterogeneous field of biological therapies in orthopedics, including blood-based, cell-based and various other approaches.

Since the first published clinical application of such a biologic therapy, the transfusion of autologous platelet-rich plasma (PRP) in cardiac surgery patients in 1987, the number of such therapies and their indications has grown substantially (1). While some, such as gene therapies, are mostly still in their pre-market phase, the number of products with regulatory clearance already achieved is growing (2, 3). Consequently, the clinical utilization of such therapies has increased in popularity as has its market (4). This is particularly the case in fields in which regenerative approaches are in high demand such as sports medicine, where over 66% of members of the American Orthopaedic Society for Sports Medicine (AOSSM) report the use of at least one OB (5, 6). However, the heterogeneous nature of OB therapies, as well as their preparation and administration protocols, poses a unique challenge regarding the creation of standardized treatment regimens (7, 8, 9). While several societies have taken on this challenge by issuing consensus statements, evidence-based guidelines are scarce (10, 11, 12, 13, 14).

This review summarizes currently existing guidelines and high-quality evidence on the clinical use of popular OBs, with a focus on blood-based and cell-based therapies currently available on the market.

## Orthobiologics

### Current Orthobiologics in practice

While a standardized nomenclature is lacking, most OB therapies currently on the market can be categorized regarding whether they are blood-based (e.g. PRP, autologous conditioned serum (ACS), or autologous protein solution (APS)) or tissue/cell-based (e.g. autologous chondrocyte implantation (ACI), bone marrow aspirate concentrate (BMAC) or mesenchymal stromal cells (MSCs)).

#### Blood-based therapies

Blood-based OBs are autologous therapies, derived from a patient’s peripheral blood and injected at the preferred site of action. Containing an extensive list of cytokines and growth factors, their mode of action was described as a limitation of inflammation and, consequently, a promotion of tissue regeneration (15, 16). Depending on whether the derived blood was subjected to further modifications regarding certain proteins, therapies are either considered PRP or PRP-like (ACS or APS) (17).

##### PRP therapies

PRP products are used in a wide array of pathologies, covering acute injuries such as rotator cuff tears as well as degenerative diseases such as knee osteoarthritis (KOA) (18, 19). With a large number of different commercial solutions for the preparation of PRP therapies available on the market, the same applies to the formulation of the injectable product regarding its concentration of platelets, leukocytes and growth factors (2, 20). To standardize therapies, several studies have reported classification systems for PRP products (21, 22, 23).

The level of evidence supporting PRP therapies varies considerably across different pathologies, with KOA, lateral epicondylitis of the elbow, and plantar fasciitis being the most extensively studied through randomized controlled trials (RCTs) (24).

A meta-analysis including 14 RCTs and a total of 1,423 patients evaluated the effects of PRP on pain and physical function in KOA (25). The authors reported significant improvements in both outcome measures – WOMAC total score and WOMAC physical function subscore at 3, 6 and 12 month post-injection in the PRP group. However, they also noted that ten of the included RCTs were at high risk of bias and 4 at moderate risk.

In the context of lateral epicondylitis, a systematic review and meta-analysis reported improvements surpassing the minimal clinically important difference in most patient-reported outcome measures (PROMs) following PRP treatment (26). While no risk of bias assessment tool was applied by the authors, they state that: ‘many source studies used in the present analysis did not include basic data such as age, sex distribution, PRP separation parameters, raw PROM values and SDs of outcomes’.

Regarding plantar fasciitis, a meta-analysis of 21 RCTs involving 1,356 patients found PRP injections to be significantly more effective in reducing pain compared to corticosteroid injections (CSI), extracorporeal shock wave therapy (ESWT) and placebo (27). In addition, PRP led to greater improvements in the American Orthopaedic Foot and Ankle Society Ankle-Hindfoot Score compared to CSI and placebo. However, no significant differences were observed between PRP and CSI, ESWT or placebo in terms of reduction of plantar fascia thickness or the foot function index. The included studies demonstrated considerable variability in PRP preparation methods, and the overall quality of the RCTs was reported as low.

##### PRP-like therapies

Equivalent to PRP, PRP-like therapies, e.g., ACS and APS are based on autologous patient blood, which is prepared and the product immediately re-injected. However, unlike PRP, their preparation includes additional steps to increase the concentration of all (APS) or certain (ACS: IL-1 receptor antagonist) components of the derived patient’s blood (28, 29). As of today, no RCTs on PRP-like therapies exist and studies on their outcome exhibit limited power (30, 31).

#### Tissue/cell-based therapies

Development pipelines for tissue/cell-based therapies mandate in-depth knowledge and expertise in basic science, the rigorous organization and design of clinical trials and of the regulatory approval process (32). Only a few therapy platforms with limited high-level evidence are in clinical use in orthopedics. High risk of bias arising from industry sponsoring and an overall poor methodological quality of conducted studies are major problems in the evaluation of the current evidence (33).

##### High-level evidence

A recent meta-analysis screened all RCTs from 2000 to 2023 that reported on intraarticular therapies for OA and recruited at least 100 participants per group. Only one study with a cell-based therapy investigating the efficacy of autologous expanded adipose-derived MSCs (ADMSC) was found (33). High risk of bias was estimated in the published data because of unclear allocation concealment and missing outcome data and standardized mean difference showed no superior effect in pain relief at 12 weeks (33). However, the original publication reported that significantly more patients in the ADMSC group reached MCID for VAS and WOMAC score at 6 months (34).

Concerning stromal vascular fraction (SVF) and BMAC, a recent large RCT with 480 patients published after the inclusion stop of the above-mentioned review could not show superiority of BMAC or SVF when compared to placebo at 12-month follow-up (35).

##### Low-level evidence

A meta-analysis with five smaller RCTs, including less than 30 patients per group per study, showed safety and efficacy for ADMSC and SVF treatment (36). However, assessment of bias was less strict as in the review by Pereira *et al.* and no study reached low risk of bias at every key domain of the Cochrane risk of bias tool. Another recent meta-analysis screening RCTs for cell therapies in OA treatment found 15 RCTs, including less than 30 patients per group per study on average. The included studies used allogeneic and autologous bone marrow-derived cells (BMSC, eight studies), ADMSC (six studies) and umbilical cord-derived cells (UCMSC, one study) and showed sufficient safety and efficacy (37). However, only six of the 15 included studies were estimated to have low risk of bias in all key domains of the assessment.

##### Conclusion

In summary, the most studied cell-based therapies for OA in clinical use are BMSC/BMAC, ADMSC/SVF variants. All above-mentioned therapies are considered safe in clinical use. However, current evidence cannot support clear mechanisms of action. Presently, this is addressed by in-depth responder/non-responder analyses and patient stratification approaches. When tissue/cell-based therapies are offered to OA patients as one treatment option, this limited high-level evidence must be communicated transparently.

### Existing guidelines for clinical applications and indications

Despite increasing clinical adoption and growing evidence supporting the efficacy of OB therapies, international guidelines remain limited and fragmented. This lack of comprehensive guidance is primarily due to significant variability and uncertainty surrounding the indications, dosages, preparation techniques, administration protocols, patient selection criteria and regulatory aspects across different regions. Notably, current international guidelines (AAOS (14) and ESSKA (11)) predominantly focus on KOA, leaving several other orthopedic indications without standardized guidance.

#### Bone healing and osteoinduction

Currently, no widely recognized or comprehensive international guidelines specifically address OB applications for bone healing or osteoinduction. A recent meta-analysis evaluated the efficacy of PRP and other adjuvant therapies for fracture nonunion and delayed union by analyzing 30 RCTs involving 1,711 patients (38). The analysis showed that combining PRP with BMAC notably improved fracture healing rates, ranking among the most effective interventions. These findings further emphasize the potential clinical utility of PRP in bone healing management; however, definitive recommendations remain limited due to ongoing variability in protocols and lack of standardized guidelines.

#### Cartilage defects and osteoarthritis

Both the AAOS (14) and ESSKA (11) consensus statements recommend using PRP primarily in patients with mild to moderate KOA (Kellgren–Lawrence grades I–III), while they remain cautious regarding its application in severe OA cases. In addition, both guidelines recognize PRP as generally safe, with minimal side effects, and advise against its use in patients with systemic infections, cancer or inflammatory joint diseases. Furthermore, AAOS and ESSKA agree that patients should avoid NSAIDs around the time of PRP injections to maximize therapeutic efficacy. Both also acknowledge that PRP offers clinical benefits, particularly in terms of pain relief and improved joint function, although evidence supporting structural improvements such as cartilage regeneration remains limited.

However, several differences exist between the two guidelines. For example, AAOS suggests injecting PRP volumes ranging from 4 to 8 mL per knee joint, whereas ESSKA allows more flexibility, recommending volumes between 2 and 12 mL without definitive guidance. Regarding treatment frequency, AAOS typically advises a course of one to three injections spaced weekly or biweekly, while ESSKA recommends two to four injections per cycle with intervals between 1 and 3 weeks. In addition, there is a notable divergence in preferences concerning PRP preparation: AAOS strongly favors leukocyte-poor PRP due to its anti-inflammatory properties, whereas ESSKA maintains a neutral stance, acknowledging the efficacy of both leukocyte-poor and leukocyte-rich PRP, and emphasizing the importance of clearly documenting platelet and leukocyte concentrations. Both emphasize the need for standardization to minimize variability.

Despite addressing cartilage repair indirectly in KOA guidelines, explicit guidelines for isolated cartilage defects in joints other than the knee are missing. A recent meta-analysis involving nine studies (315 patients) evaluating intra-articular use of MSCs, PRP and hyaluronic acid for cartilage and osteochondral defects in early-stage OA indicated improvements in Lysholm, VAS, KSS and WOMAC scores (39). However, the investigated studies have been inhomogeneous and in parts biased, which is why these findings have not yet been incorporated into any established clinical guidelines.

#### Ligament repair and tendon injury

Although clinical practice increasingly utilizes PRP and tissue/cell-based OBs to manage ligament and tendon injuries, current consensus statements have not provided specific recommendations or standardized guidelines addressing these conditions explicitly. Numerous systematic reviews have assessed these therapies, yet a definitive meta-analysis is still unavailable due to high heterogeneity among indications and studies, highlighting the need for more standardized research approaches.

#### Osteotomies

A recent meta-analysis evaluated the clinical efficacy and safety of intra- and postoperative intra-articular PRP injections in the context of high tibial osteotomy (HTO) as a treatment for KOA (40). The study analyzed ten studies (four RCTs and six cohort studies) including 625 patients. The results demonstrated significant improvements in pain relief (VAS score), knee function (WOMAC, Lysholm scores and range of motion) and cartilage repair outcomes compared to controls (HTO alone, PRP alone or other treatments). However, there are no standardized or widely adopted guidelines that detail the specific indications, protocols or dosages for the intra- or postoperative intra-articular use of OBs in conjunction with osteotomies.

#### Meniscus lesions

A recent meta-analysis included nine studies (two RCTs and seven non-RCTs) with a total of 1,164 patients (41). It compared meniscus repair outcomes augmented with and without PRP. The results demonstrated a significantly lower meniscus repair failure rate and better postoperative pain control (VAS scores) in the PRP group compared to controls. However, no significant differences were found regarding functional outcomes measured by IKDC and Lysholm scores. The authors concluded that despite promising results regarding failure rates and pain management, current evidence from RCTs remains insufficient to fully support routine PRP use for enhancing functional outcomes following meniscus repair. The existing consensus does not provide explicit recommendations for OB applications in meniscal injuries.

#### Spine

Currently, no established clinical guidelines exist regarding the use of OBs in spinal pathologies. A recent meta-analysis included three RCTs involving a total of 131 patients evaluating PRP injections to the lumbar facet/sacroiliac joint or the intervertebral disk for the treatment of lower back pain (42). Compared with control interventions, PRP injections significantly reduced pain scores, increased the number of patients experiencing more than 50% pain relief at 3 months and provided relatively higher patient satisfaction. Furthermore, PRP injections were not associated with an increased risk of adverse events. Another recent meta-analysis evaluated PRP specifically in postoperative spinal surgery settings (43). This analysis included seven studies (two RCTs, two prospective and three retrospective studies) with a total of 299 patients (150 PRP and 149 placebo). The results showed that intraoperative PRP injections combined with posterior or posterolateral lumbar fusion (PLF) surgery reduced postoperative back pain and accelerated bone union time compared to PLF alone, though no significant differences were observed in overall fusion rates or adverse events.

#### Conclusion

Existing international guidelines remain heavily focused on KOA management, particularly with PRP, while neglecting other significant orthopedic conditions and applications. The clinical community urgently requires low-risk-of-bias studies and robust data to fill comprehensive guidelines addressing a wider range of orthopedic applications, including bone healing, ligament repair, tendon injury, osteotomies and meniscal lesions. Such guidelines should be based on rigorous standardization, substantial clinical evidence and clear regulatory frameworks to optimize patient care and outcomes.

### Challenges in clinical adoption and the creation of guidelines

Several reviews have criticized the high variability among preparation and treatment protocols, as well as the lack of data on the quality of therapies administered among studies investigating the outcome of OB therapies (7, 8, 17, 44, 45, 46). Furthermore, with a vast range of indications, a wide array of scoring systems is being used to monitor clinical outcomes. This heterogeneity poses a significant bias to the synthesis of results in large-scale meta-analyses.

Apart from research-related obstacles regarding the creation of guidelines, challenges in clinical adoption are often regulatory-related. The clearance and even more, the approval of therapies by authorities can be a complex process, potentially limiting the accessibility to and cost-effectiveness of OB treatments (47).

However, an understanding of the various challenges in the creation of guidelines and clinical adoption of OB therapies, as well as interdisciplinary platforms fostering exchange and collaboration between researchers, clinicians and industry are valuable tools in overcoming these obstacles.

### Future perspectives

Beyond existing blood- and cell-based therapies, novel OB strategies are being explored to further enhance regenerative potential. One promising avenue is the use of exosome-based therapies, which have gained significant attention due to their regenerative and immunomodulatory properties. Exosomes are nanosized (40–150 nm) extracellular vesicles of endosomal origin, released by the fusion of multivesicular bodies with the plasma membrane. Secreted by various cell types, including MSCs, they serve as key mediators of intercellular communication through their bioactive cargo of proteins, cytokines, microRNAs and lipids. This cargo enables exosomes to modulate inflammation, support tissue repair and influence extracellular matrix remodeling. Unlike live cell therapies, exosome-based approaches offer a cell-free alternative with lower immunogenic risk and reduced concerns regarding tumorigenicity or uncontrolled proliferation (48). Although preliminary studies suggest that exosome therapy may be a viable option for cartilage repair and OA treatment, large-scale clinical trials are still required to establish their efficacy and safety.

In parallel, advances in gene-based therapies and mRNA-based approaches have opened new possibilities for musculoskeletal regeneration. The development of CRISPR-based gene-editing technologies allows for precise modifications in chondrogenesis and osteogenesis, potentially enhancing the effectiveness of cell-based therapies (49). Similarly, mRNA therapeutics, which have gained prominence in vaccine development, could be leveraged to stimulate endogenous repair mechanisms within musculoskeletal tissues (50). Despite their potential, these strategies remain in early experimental stages with significant challenges related to delivery mechanisms, durability of effects and regulatory approval still to be addressed.

Another critical area of future research is the stratification of patients to identify responders and non-responders to OB therapies. One of the major limitations of current treatments is the variability in patient response. This unpredictability underscores the need for biomarker-based stratification, incorporating genetic, proteomic and metabolomic profiling to predict treatment efficacy on an individual level. Recent findings suggest that individual immune cell compositions and cytokine profiles play a critical role in determining the biological activity and efficacy of OB therapies. For instance, variability in PRP composition has been shown to be largely influenced by the donor’s immune profile, including the presence of specific cytokines and immune cell subtypes (51). The development of predictive models based on immune and molecular markers will be essential in guiding treatment decisions, ensuring that only patients with a high likelihood of response receive these often costly and complex therapies.

To fully realize the potential of OB, standardization of treatment protocols is imperative. Heterogeneity in cell preparation techniques, dosing strategies and administration methods has resulted in inconsistent clinical outcomes and remains a major barrier to regulatory approval. The establishment of universally accepted preparation and application methods will be crucial for optimizing clinical efficacy and facilitating broader adoption.

Bridging the gap between experimental findings and clinical implementation requires ongoing high-quality, multi-center clinical trials. Examples of structured translational research in the field can be found in European-funded consortia such as the PROTO consortium, which investigates a placenta-derived allogeneic cell therapy for mid-stage OA (52). This approach aims to modulate the joint microenvironment, potentially delaying disease progression and offering a disease-modifying alternative to symptomatic treatments.

The future of OBs lies in refining existing therapies, integrating novel regenerative technologies and personalizing treatment strategies. The emergence of exosome-based therapies, gene-editing approaches and biomarker-driven patient stratification offers exciting new possibilities for musculoskeletal regeneration. However, the success of these innovations will depend on their rigorous evaluation through standardized clinical protocols and well-structured clinical trials.

## Conclusion

International guidelines on the use of OBs supported by high-quality evidence remain limited. The availability of RCTs varies widely across products and pathologies and many studies carry a considerable risk of bias. Future efforts should aim to establish clear standards for the indication, preparation, application and outcome reporting of OB therapies to fully realize their clinical potential.

## ICMJE Statement of Interest

The authors declare that they have no conflicts of interest in the authorship and publication of this contribution.

## Funding Statement

This study was partly funded by the DFG Collaborative Research Center 1444, German Federal Ministry of Education and Research (Grant: 031L0234B) and the European Union under Grant Agreement Nr. 101095635 (PROTO). Views and opinions expressed are those of the authors only and do not necessarily reflect those of the European Union or the European Health and Digital Executive Agency (HADEA). Neither the European Union nor the granting authority can be held responsible for them. A H is a participant in the BIH Charité Junior Clinician Scientist Program funded by the Charité – Universitätsmedizin Berlin and the Berlin Institute of Health at Charité (BIH).

## Author contribution statement

Each named author has substantially contributed to conducting the underlying research and drafting this manuscript.

## Ethics

The review was conducted in accordance with the Declaration of Helsinki.
